# Antioxidants and clinical outcomes of patients with coronavirus disease 2019: A systematic review of observational and interventional studies

**DOI:** 10.1002/fsn3.3034

**Published:** 2022-09-02

**Authors:** Sahar Foshati, Fatemeh Mirjalili, Mahsa Rezazadegan, Farnoosh Fakoorziba, Reza Amani

**Affiliations:** ^1^ Department of Clinical Nutrition, School of Nutrition and Food Science, Food Security Research Center Isfahan University of Medical Sciences Isfahan Iran; ^2^ Marquise Hospitality, Compass Group Mississauga Ontario Canada

**Keywords:** antioxidants, ascorbic acid, COVID‐19, oxidative stress, SARS‐CoV‐2, selenium, vitamin D, zinc

## Abstract

Coronavirus disease 2019 (COVID‐19) is a newly emerging viral infection caused by severe acute respiratory syndrome coronavirus 2 (SARS‐CoV‐2). Oxidative stress appears to be a prominent contributor to the pathogenicity of SARS‐CoV‐2. Therefore, we carried out a systematic review of human observational and interventional studies to investigate the role of some antioxidants such as vitamins A, E, D, and C, selenium, zinc, and α‐lipoic acid in the main clinical outcomes of subjects with COVID‐19. Google Scholar, Cochrane Library, Web of Science, Scopus, and Medline were searched using Medical Subject Headings (MeSH) and non‐MeSH terms without restrictions. Finally, 36 studies for vitamins C and D, selenium, and zinc were included in this systematic review; however, no eligible studies were found for vitamins A and E as well as α‐lipoic acid. The results showed the promising role of vitamin C in inflammation, Horowitz index, and mortality; vitamin D in disease manifestations and severity, inflammatory markers, lung involvement, ventilation requirement, hospitalization, intensive care unit (ICU) admission, and mortality; selenium in cure rate and mortality; and zinc in ventilation requirement, hospitalization, ICU admission, biomarkers of inflammation and bacterial infection, and disease complications. In conclusion, it seems that antioxidants, especially vitamins C and D, selenium, and zinc, can improve multiple COVID‐19 clinical outcomes. Nevertheless, more studies are necessary to affirm these results.

## Graphical abstract

The results showed the beneficial role of vitamin C in inflammation, Horowitz index, and mortality; vitamin D in disease manifestations and severity, inflammatory markers, lung involvement, ventilation requirement, hospitalization, intensive care unit (ICU) admission, and mortality; selenium in cure rate and mortality; and zinc in ventilation requirement, hospitalization, ICU admission, biomarkers of inflammation and bacterial infection, and disease complications.

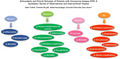

## 1 INTRODUCTION

Coronavirus disease 2019 (COVID‐19) is a viral infection caused by a highly contagious pathogen named severe acute respiratory syndrome coronavirus 2 (SARS‐CoV‐2). This disease usually results in mild to moderate influenza‐like symptoms, including fever, dry cough, and fatigue but can also lead to serious outcomes, such as hypoxemia, dyspnea, chest pain, and death. In early 2020, COVID‐19 infection speedily spread throughout the world and was announced as a global pandemic by the World Health Organization (WHO; Budholiya et al., 2020). Currently, there are options for COVID‐19 management. Some antiviral medications and monoclonal antibodies may be effective in the treatment of COVID‐19 (Takashita et al., 2022). In addition, various vaccines containing nonreplicating viral vector, protein subunit, RNA, DNA, and inactivated virus are approved and available for the prevention of COVID‐19 (Kudlay & Svistunov, 2022). Nevertheless, in spite of all medical advances in recent years, this infectious disease is still posing significant threats to public health as well as global economy. Therefore, it is inevitable to identify different strategies for the management of COVID‐19.

Oxidative stress, an imbalance between prooxidants and antioxidants in favor of the former, has been proposed as a prominent contributor to pathogenicity of SARS‐CoV‐2 (Cecchini & Cecchini, 2020). It seems that this virus binds to angiotensin‐converting enzyme (ACE) 2, disturbs the renin‐angiotensin system (RAS), and causes oxidative stress in the body (Silvagno et al., 2020). In addition, although SARS‐CoV‐2 can affect human beings of all ages, individuals with already elevated levels of oxidative stress including those with old age, obesity, cardiovascular disease, or diabetes mellitus have been reported to be at greater risk for severe COVID‐19 (Zhou et al., 2020). It seems that oxidative stress per se can weaken the immune system, induce viral activation, stimulate the production of proinflammatory chemokines and cytokines, and lead to inflammation and cell death in people with COVID‐19 (Chernyak et al., 2020; Delgado‐Roche & Mesta, 2020). Given these evidences, oxidative stress may have a role in the incidence, severity, and mortality of COVID‐19, and therefore, antioxidants could be a potential intervention to control COVID‐19.

**FIGURE 1:**
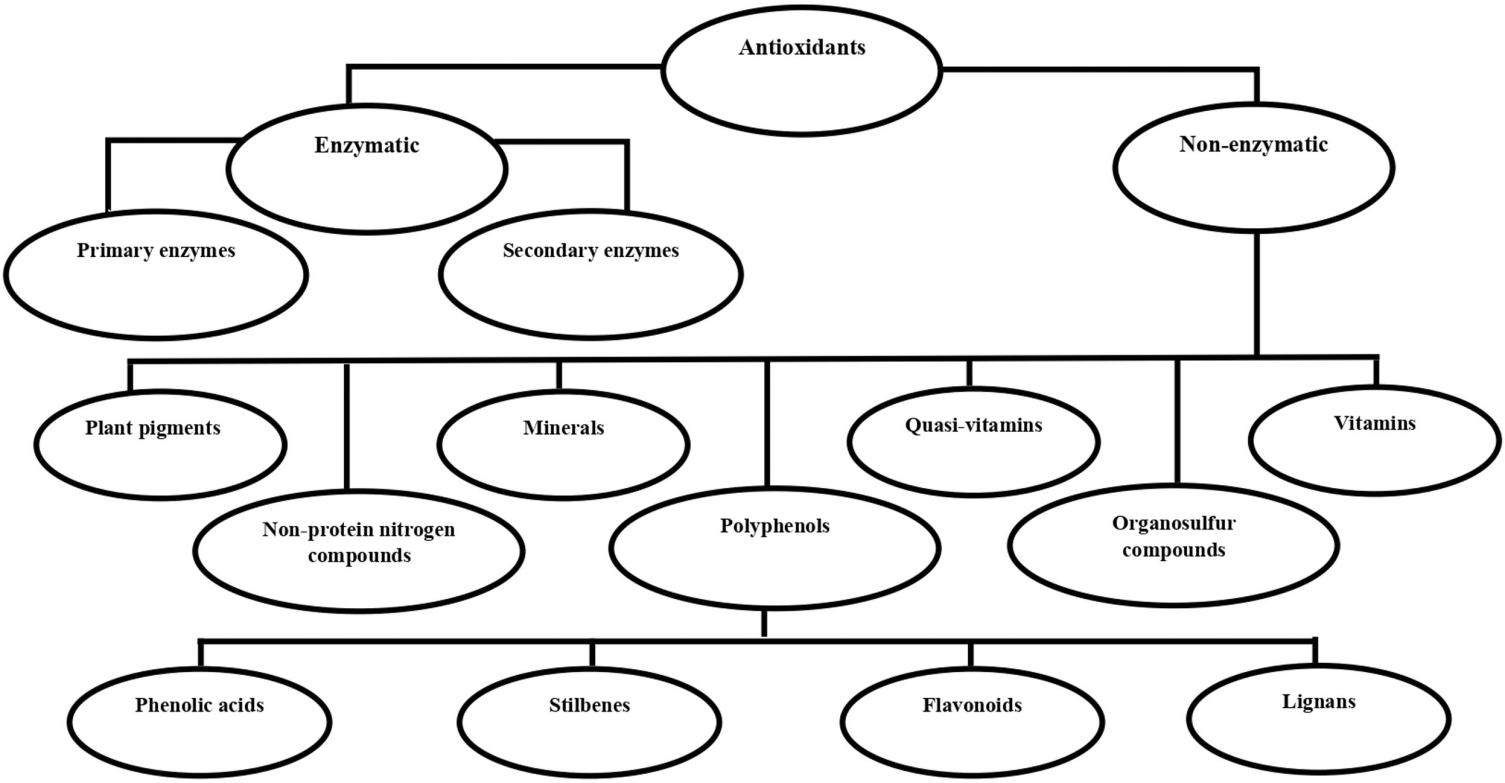
Classification of antioxidants

Antioxidants are endogenous or exogenous substances that prevent, delay, or repair oxidative damage to biological macromolecules (Halliwell, 2007). As shown in Figure 1, these beneficial substances are classified into two groups based on the presence or absence of enzymatic activity. In the group of enzymatic antioxidants, glutathione peroxidase, catalase, superoxide dismutase, and peroxiredoxins are present as examples of primary enzymes, and glutathione reductase, glucose‐6‐phosphate dehydrogenase, and glutathione S‐transferases are present as examples of secondary enzymes (Mehta & Gowder, 2015; Nimse & Pal, 2015; Ratnam et al., 2006). In the group of nonenzymatic antioxidants, there are multiple subgroups that are mainly derived from dietary sources. These subgroups include vitamins (e.g., vitamins A, D, E, and C), minerals (e.g., zinc and selenium), quasi‐vitamins (e.g., α‐lipoic acid), plant pigments (e.g., carotenoids), organosulfur compounds (e.g., allyl sulfide), nonprotein nitrogen compounds (e.g., histidine), and polyphenols (e.g., ellagic acid; Carocho & Ferreira, 2013; Sharma et al., 2018; Zhang & Tsao, 2016). Generally, antioxidants can inhibit the generation of free radicals, quench singlet oxygen, interrupt the propagation of autoxidation chain reactions, convert hydroperoxides or metal prooxidants into stable products, suppress prooxidative enzymes, and enhance immune system (Rajendran et al., 2014). It is interesting that they may even function as antiviral agents. For instance, some polyphenols have high binding affinity for pivotal proteins of SARS‐CoV‐2 such as spike protein, RNA‐dependent RNA polymerase, papain‐like protease, and 3‐chymotrypsin‐like protease (Paraiso et al., 2020). Importantly, these pivotal proteins are involved in the host cell recognition, transcription, and replication of SARS‐CoV‐2 (Cannalire et al., 2020).

All in all, antioxidants may be helpful for individuals infected with SARS‐CoV‐2. Therefore, we carried out a systematic review of human observational and interventional studies to investigate the role of some important antioxidants in major clinical outcomes of subjects with COVID‐19. In the present review, we focused on six well‐known antioxidants including vitamin A, vitamin E, vitamin C, zinc, selenium, and α‐lipoic acid as well as one vitamin with recently discovered antioxidant property, i.e., vitamin D.

## 2 METHODS

### 2.1 Research question

This review was performed according to the guidelines available for systematic reviews of observational (Stroup et al., 2000) and interventional studies (Page et al., 2021). The PECO/PICO approach (participants, exposure/intervention, comparator, and outcome) was used to define the review question (Morgan et al., 2018; Santos et al., 2007). The participants of the studies included in this systematic review were individuals with COVID‐19, regardless of their age. In observational studies, different dietary/supplement intake or biological sample levels of vitamins A, C, D, or E, selenium, zinc, or α‐lipoic acid were compared with each other. In interventional studies, supplementation with vitamins A, C, D, or E, selenium, zinc, or α‐lipoic acid was compared to matched placebo, control group, or none (i.e., without comparator). The outcomes of this systematic review were clinical outcomes of COVID‐19 reported in the included studies (e.g., disease severity, disease manifestations and complications, inflammatory biomarkers, hospitalization, and mortality).

### 2.2 Search strategy

Google Scholar, Cochrane Library, Web of Science, Scopus, and Medline were searched up to January 11, 2021. The literature search was done using the following Medical Subject Headings (MeSH) and non‐MeSH terms without restrictions: (“vitamin A" OR “retinoid” OR “retinol” OR “retinal” OR “retinoic acid" OR “retinyl ester” OR “aquasol A" OR “vitamin C" OR “ascorbic acid" OR “ascorbate” OR “vitamin D" OR “cholecalciferol” OR “colecalciferol” OR “ergocalciferol” OR “calciferol” OR “hydroxycholecalciferol” OR “calcifediol” OR “25‐hydroxyvitamin D" OR “calcidiol” OR “25‐hydroxycholecalciferol” OR “calcitriol” OR “1,25‐dihydroxyvitamin D” OR “1,25‐dihydroxycholecalciferol” OR “alfacalcidol” OR “1‐alpha‐hydroxyvitamin D” OR “1‐α‐hydroxyvitamin D" OR “paricalcitol” OR “vitamin E" OR “tocopherol” OR “tocotrienol” OR “selenium” OR “selenite” OR “selenate” OR “Se” OR “zinc” OR “Zn” OR “thioctic acid" OR “alpha‐lipoic acid" OR “α‐lipoic acid" OR “lipoic acid") AND (“COVID‐19” OR “coronavirus disease‐19” OR “coronavirus disease 2019” OR “2019‐nCoV” OR “2019 novel coronavirus” OR “SARS‐CoV‐2” OR “severe acute respiratory syndrome coronavirus 2” OR “Wuhan coronavirus”). The full search strategy for each database or search engine is presented in Table S1.

### 2.3 Study selection

The study selection was done by two independent authors. First, the title and abstract of publications were screened to find pertinent ones. Second, the full text of pertinent articles was screened to discern eligible observational and interventional studies. The inclusion criteria for observational studies were: (1) studies with observational design (cross‐sectional, case–control, cohort, and ecological study); (2) studies conducted on COVID‐19 patients; (3) studies considered different levels of vitamins A, C, D, or E, selenium, zinc, or α‐lipoic acid in eaten foods/supplements or biological samples as the exposure of interest; (4) studies considered any clinical outcomes of COVID‐19 (e.g., disease severity, disease manifestations and complications, inflammatory biomarkers, hospitalization, and mortality) as the outcomes of interest; and (5) studies reported the relationship between the exposure and outcome of interest in the form of odds ratio, relative risk, hazard ratio, β or B coefficient, or correlation coefficient. The inclusion criteria for interventional studies were: (1) studies with interventional design (pre/post trial, quasi‐experimental trial, and randomized controlled trial); (2) studies performed on COVID‐19 patients; and (3) studies investigated the effect of infusion or oral supplementation with vitamins A, C, D, or E, selenium, zinc, or α‐lipoic acid on any clinical outcomes of COVID‐19. The exclusion criteria for observational and interventional studies were: (1) observational studies reported the percentage, median, or mean of the above outcome of interest in different subgroups of the above exposure of interest or vice versa (e.g., descriptive studies); (2) observational studies considered sunlight exposure, an imprecise estimate of vitamin D status (McCarty, 2008), as the exposure variable; (3) observational studies considered the incidence of COVID‐19 as the outcome variable; (4) interventional studies administered one of the aforesaid seven antioxidants in combination with other nutrients; (5) studies reported duplicate data; (6) studies written in non‐English languages; and (7) studies published as preprints (not peer‐reviewed articles), perspectives, commentaries, editorials, letters, reviews, conference reports, case reports, study protocols, in vitro or ex vivo experiments, and animal models.

### 2.4 Data extraction

General characteristics of observational and interventional researches were extracted and tabulated by two independent investigators using the Cochrane data collection form. These characteristics include author names, year of publication, study design and location, mean age of patients, total and gender‐specific sample size, exposure, intervention and control groups, and main results of COVID‐19 clinical outcomes. It is worth mentioning that the level of agreement between investigators for data collection was appropriate (Kappa = 0.81).

### 2.5 Quality assessment

The quality assessment of studies was conducted by two independent reviewers using the Academy of Nutrition and Dietetics Quality Criteria Checklist (QCC) for Primary Research (Academy of Nutrition and Dietetics, 2016). The quality of each study was rated as positive, neutral, or negative according to the applicability of findings to practice, and 10 validity questions regarding research question, participant selection and withdrawal, intervention/exposure, comparison, study blinding, the reliability of outcome measures, statistical analysis, study limitations, and funding sources. The level of agreement between reviewers for quality assessment was very good (Kappa = 0.92).

## 3 RESULTS

### 3.1 Literature search

**FIGURE 2:**
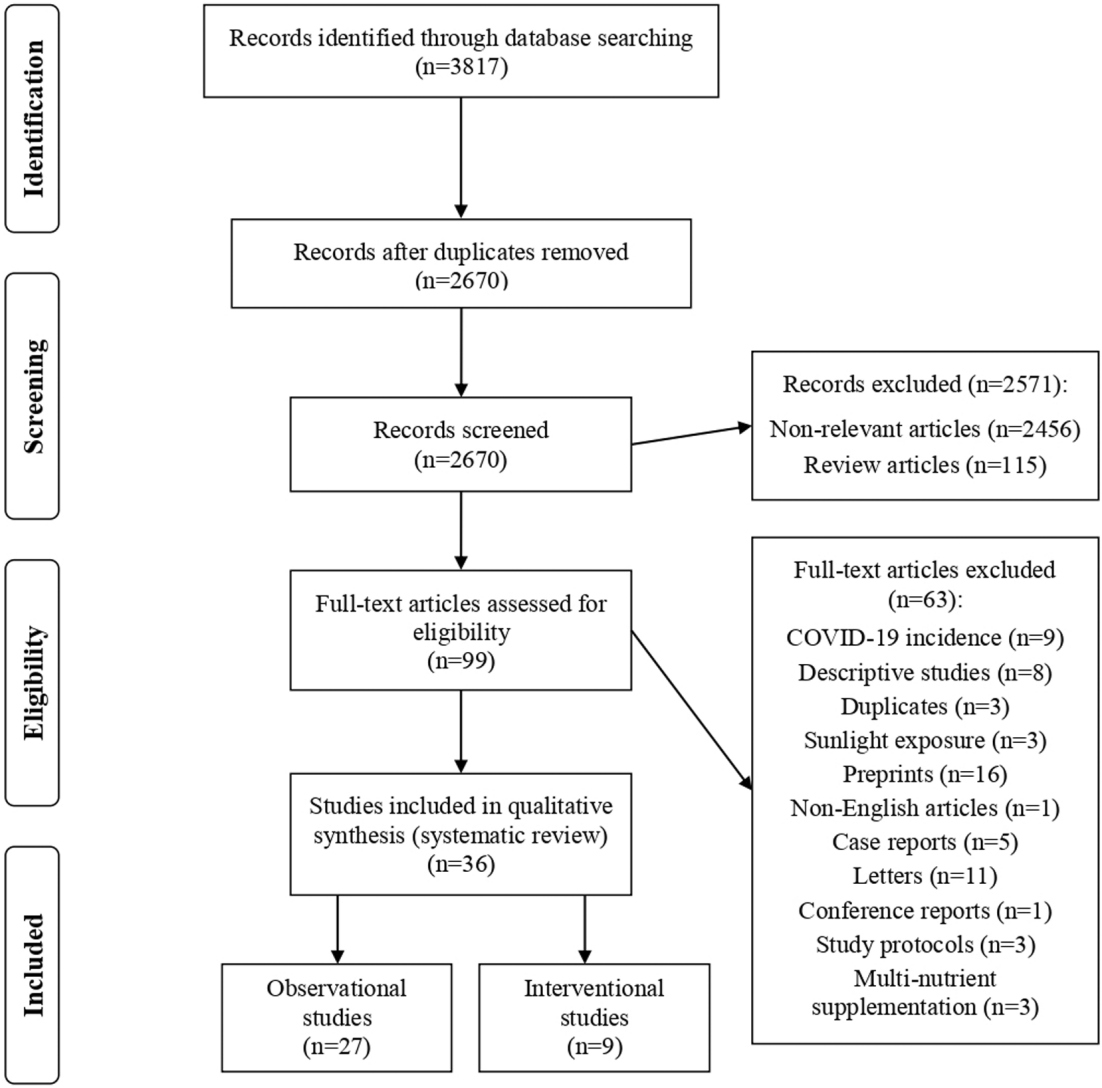
Flow diagram of the study selection process

As presented in Figure 2, 3817 publications were retrieved through searching four online databases and one search engine. After excluding duplicate publications, 2670 reports were left for screening. In primary screening, 2571 papers were removed based on title and abstract. In secondary screening, 63 papers were removed based on full text. Lastly, a total of 36 studies, 27 observational (Abrishami et al., 2021; Anuk et al., 2021; Arvinte et al., 2020; Bagheri et al., 2020; Baktash et al., 2021; Carpagnano et al., 2021; Daneshkhah et al., 2020; De Smet et al., 2021; Hars et al., 2020; Heller et al., 2020; Hernández et al., 2021; Jothimani et al., 2020; Karahan & Katkat, 2021; Laird et al., 2020; Luo et al., 2021; Maghbooli et al., 2020; Merzon et al., 2020; Moghaddam et al., 2020; Padhi et al., 2020; Panagiotou et al., 2020; Pizzini et al., 2020; Radujkovic et al., 2020; Singh et al., 2020; Yasui et al., 2020; Ye et al., 2021; Yılmaz & Şen, 2020; Zhang et al., 2020) and 9 interventional (Annweiler, Corvaisier, et al., 2020; Annweiler, Hanotte, et al., 2020; Carlucci et al., 2020; Castillo et al., 2020; Derwand et al., 2020; Hiedra et al., 2020; Rastogi et al., 2022; Yao et al., 2021; Zhang et al., 2021) studies, were included in the current systematic review.

### 3.2 Study characteristics and quality

The characteristics and quality of observational and interventional studies are summarized in Tables 1 and 2, respectively. The included studies were published between 2020 and 2022. According to the time of conducting the studies, the following SARS‐CoV‐2 variants are covered in this systematic review: Alpha, Beta, Gamma, Delta, Epsilon, Zeta, Iota, and Kappa (World Health Organization, 2022). The included studies were performed in USA (*n* = 5; Arvinte et al., 2020; Carlucci et al., 2020; Derwand et al., 2020; Hiedra et al., 2020; Yao et al., 2021), China (*n* = 4; Luo et al., 2021; Ye et al., 2021; Zhang et al., 2020, 2021), Germany (*n* = 3; Heller et al., 2020; Moghaddam et al., 2020; Radujkovic et al., 2020), Iran (*n* = 3; Abrishami et al., 2021; Bagheri et al., 2020; Maghbooli et al., 2020), India (*n* = 3; Jothimani et al., 2020; Padhi et al., 2020; Rastogi et al., 2022), Turkey (*n* = 3; Anuk et al., 2021; Karahan & Katkat, 2021; Yılmaz & Şen, 2020), multi‐countries (*n* = 3; Daneshkhah et al., 2020; Laird et al., 2020; Singh et al., 2020), UK (*n* = 2; Baktash et al., 2021; Panagiotou et al., 2020), France (*n* = 2; Annweiler, Corvaisier, et al., 2020; Annweiler, Hanotte, et al., 2020), Spain (*n* = 2; Castillo et al., 2020; Hernández et al., 2021), Austria (*n* = 1; Pizzini et al., 2020), Belgium (*n* = 1; De Smet et al., 2021), Italy (*n* = 1; Carpagnano et al., 2021), Switzerland (*n* = 1; Hars et al., 2020), Israel (*n* = 1; Merzon et al., 2020), and Japan (*n* = 1; Yasui et al., 2020). All studies, except one which was done only on women (Anuk et al., 2021), were conducted on both sexes. Also, all studies, except one which was performed on children (Yılmaz & Şen, 2020), were carried out on adults (≥18 years old). The included studies were designed as cross‐sectional (*n* = 6; Bagheri et al., 2020; Heller et al., 2020; Luo et al., 2021; Maghbooli et al., 2020; Moghaddam et al., 2020; Panagiotou et al., 2020), retrospective cohort (*n* = 6; Abrishami et al., 2021; Carpagnano et al., 2021; De Smet et al., 2021; Hars et al., 2020; Karahan & Katkat, 2021; Merzon et al., 2020), prospective cohort (*n* = 5; Arvinte et al., 2020; Baktash et al., 2021; Pizzini et al., 2020; Radujkovic et al., 2020; Yasui et al., 2020), case–control (*n* = 5; Anuk et al., 2021; Hernández et al., 2021; Jothimani et al., 2020; Ye et al., 2021; Yılmaz & Şen, 2020), ecological study (*n* = 5; Daneshkhah et al., 2020; Laird et al., 2020; Padhi et al., 2020; Singh et al., 2020; Jinsong Zhang et al., 2020), quasi‐experimental trial (*n* = 5; Annweiler, Corvaisier, et al., 2020; Annweiler, Hanotte, et al., 2020; Carlucci et al., 2020; Derwand et al., 2020; Yao et al., 2021), randomized controlled trial (*n* = 3; Castillo et al., 2020; Rastogi et al., 2022; Zhang et al., 2021), and pre/post trial (*n* = 1; Hiedra et al., 2020). All studies were rated as neutral quality except three that were rated as positive quality (Castillo et al., 2020; Rastogi et al., 2022; Zhang et al., 2021).

**TABLE 1:** Overview of the observational studies included in the systematic review.

Study (year)	Location	Sample size (male/female)	Mean age (years)	Design	Exposure	Main findings	Study quality^a^
Vitamin C							
Arvinte et al. (2020)	USA	21 (15/6)	61	Prospective cohort	Serum vitamin C levels	Mortality (Ø)	Neutral
Vitamin D							
Luo et al. (2021)	China	895 (405/490)	55	Cross‐sectional	˂12 ng/ml serum 25(OH)D levels	Disease severity (+), Length of hospitalization (Ø)	Neutral
De Smet et al. (2021)	Belgium	186 (109/77)	69	Retrospective cohort	<20 ng/ml serum 25(OH)D levels	Mortality (+)	Neutral
Bagheri et al. (2020)	Iran	510 (NR)	51	Cross‐sectional	Vitamin D_3_ supplement intake	Disease severity (−), Hospitalization (Ø)	Neutral
Abrishami et al. (2021)	Iran	73 (47/26)	55	Retrospective cohort	Serum 25(OH)D level <25 ng/ml serum 25(OH)D level	Lung involvement (−) Mortality (+), Survival (−)	Neutral
Carpagnano et al. (2021)	Italy	42 (30/12)	65	Retrospective cohort	<10 ng/ml serum 25(OH)D levels	Mortality (+), Survival (−)	Neutral
Hars et al. (2020)	Switzerland	160 (65/95)	86	Retrospective cohort	<20 ng/ml serum 25(OH)D levels	Mortality in males (+), Mortality in females (Ø), Survival in males (−), Survival in females (Ø)	Neutral
Karahan & Katkat (2021)	Turkey	149 (81/68)	63	Retrospective cohort	Serum 25(OH)D levels	Mortality (−), CRP (−), Neutrophil count (−), Lymphocyte count (−)	Neutral
Ye et al. (2021)	China	142 (55/87)	42	Case–control	<20 ng/ml serum 25(OH)D levels Serum 25(OH)D levels	Disease severity (+) Disease severity (−)	Neutral
Yılmaz & Şen (2020)	Turkey	85 (46/39)	7	Case–control	Serum 25(OH)D levels	Fever (−)	Neutral
Baktash et al. (2021)	UK	105 (57/48)	81	Prospective cohort	≤12 ng/ml serum 25(OH)D levels	Mortality (Ø), Chest radiological findings (Ø), Ventilation requirement (+)	Neutral
Hernández et al. (2021)	Spain	413 (253/160)	60	Case–control	Serum 25(OH)D levels <20 ng/ml serum 25(OH)D levels	D‐dimer (−), Ferritin (−), CRP (Ø), IL‐6 (Ø), Disease severity (Ø) Disease severity (Ø)	Neutral
Maghbooli et al. (2020)	Iran	235 (144/91)	59	Cross‐sectional	<30 ng/ml serum 25(OH)D levels	Disease severity (+), CRP (+), Lymphocytopenia (+), Hypoxia (+), Unconsciousness (+)	Neutral
Panagiotou et al. (2020)	UK	134 (73/61)	68	Cross‐sectional	Serum 25(OH)D levels	Increased oxygen requirements (Ø), NEWS‐2 (Ø), Chest radiological findings (Ø), CRP (Ø), Mortality (Ø)	Neutral
Merzon et al. (2020)	Israel	7807 (3234/4573)	41	Retrospective cohort	<30 ng/ml plasma 25(OH)D levels	Hospitalization (+)	Neutral
Padhi et al. (2020)	India	27 state/union territories	NR	Ecological study	Mean blood 25(OH)D levels	Mortality (−)	Neutral
Radujkovic et al. (2020)	Germany	185 (95/90)	62	Prospective cohort	<12 ng/ml serum 25(OH)D levels <20 ng/ml serum 25(OH)D levels	Disease severity (+), Mortality (+), Survival (−) Disease severity (+), Mortality (+), Survival (−)	Neutral
Pizzini et al. (2020)	Austria	109 (65/44)	58	Prospective cohort	Serum 25(OH)D levels at disease onset Serum 25(OH)D levels at follow‐up	CRP (Ø), IL‐6 (Ø), Ferritin (Ø), D‐dimer (+) CRP (Ø), IL‐6 (Ø), Ferritin (Ø), D‐dimer (Ø)	Neutral
Daneshkhah et al. (2020)	Asian, American & European countries	3 Asian, 1 American & 6 European countries	NR	Ecological study	Mean blood 25(OH)D levels	Mortality (−), hs‐CRP (−)	Neutral
Laird et al. (2020)	European countries	12 European countries	NR	Ecological study	Mean blood 25(OH)D levels	Mortality (−)	Neutral
Singh et al. (2020)	European countries	20 European countries	NR	Ecological study	Mean blood 25(OH)D levels	Mortality (Ø)	Neutral
Selenium							
Zhang et al. (2020)	China	17 cities	NR	Ecological study	Mean hair selenium levels	Cure rate (+)	Neutral
Moghaddam et al. (2020)	Germany	33 (14/19)	77	Cross‐sectional	Serum selenium levels Serum SELENOP levels Serum GPx3 activity	Mortality (−) Mortality (−) Mortality (−)	Neutral
Zinc							
Yasui et al. (2020)	Japan	62 (34/28)	52	Prospective cohort	Serum zinc levels	Disease severity (−)	Neutral
Heller et al. (2020)	Germany	35 (16/19)	77	Cross‐sectional	Serum zinc levels	Survival (+)	Neutral
Anuk et al. (2021)	Turkey	200 (0/200)	27	Case–control	Serum zinc levels	Disease severity (Ø), IL‐6 (−), ESR (−), PCT (−), BUN (+), CRP (−)	Neutral
Jothimani et al. (2020)	India	92 (NR)	33	Case–control	<80 μg/dl serum zinc levels	Disease complications (+), Corticosteroid use (+), Length of hospitalization (Ø), ICU admission (Ø), Mortality (Ø)	Neutral
Bagheri et al. (2020)	Iran	510 (NR)	51	Cross‐sectional	Zinc supplement intake	Disease severity (Ø), Hospitalization (Ø)	Neutral

*Note*: (+), direct relationship; (−), inverse relationship; (Ø), no relationship.

Abbreviations: BUN, blood urea nitrogen; CRP, C‐reactive protein; ESR, erythrocyte sedimentation rate; GPx3, glutathione peroxidase‐3; hs‐CRP, high‐sensitivity C‐reactive protein; ICU, intensive care unit; IL‐6, interleukin‐6; NEWS‐2, national early warning score 2; NR, not reported; PCT, procalcitonin; SELENOP, selenoprotein P.

^a^ Based on the Academy of Nutrition and Dietetics Quality Criteria Checklist (QCC) for Primary Research.

**TABLE 2:** Overview of the interventional studies included in the systematic review.

Study (year)	Location	Sample size (male/female)	Mean age (years)	Design	Intervention	Control	Main findings	Study quality^a^
Vitamin C								
Zhang et al. (2021)	China	56 (36/20)	67	Parallel double‐blind randomized controlled trial	24 g/d IV vitamin C for 7 days	Matched placebo	↔ IMVFD28, ↓ Mortality, ↔ Length of hospitalization, ↔ Disease complications, ↔ SOFA, ↑ PaO_2_/FiO_2_, ↓ IL‐6, ↔ MAP, ↓ TBIL, ↔ WBC, ↔ PCT, ↔ CRP, ↔ Cr, ↔ BUN, ↔ PT	Positive
Hiedra et al. (2020)	USA	17 (10/7)	64	Pre‐post trial	3 g/d IV vitamin C for 3 days	–	↓ D‐dimer, ↓ Ferritin, ↔ FiO_2_	Neutral
Vitamin D								
Castillo et al. (2020)	Spain	76 (45/31)	53	Parallel randomized controlled trial	Oral calcifediol with dose of 0.532 mg at hospital admission, 0.266 mg on 3th and 7th day, and 0.266 mg/wk until discharge or ICU admission	Control group	↓ ICU admission	Positive
Annweiler, Corvaisier et al. (2020)	France	77 (39/38)	88	Quasi‐experimental trial	Oral vitamin D_3_ with dose of 50,000 IU/mo or 80,000–100,000 IU/2–3 mo over the preceding year 80,000 IU oral vitamin D_3_ after COVID‐19 diagnosis	Control group	↓ Mortality, ↓ OSCI, ↑ Survival ↔ Mortality, ↔ OSCI, ↔ Survival	Neutral
Rastogi et al. (2022)	India	40 (20/20)	49	Parallel randomized controlled trial	60,000 IU/d oral vitamin D_3_ for 7 days	Matched placebo	↓ Fibrinogen, ↔ D‐dimer, ↔ CRP, ↔ PCT, ↔ Ferritin	Positive
Annweiler, Hanotte, et al. (2020)	France	66 (15/51)	88	Quasi‐experimental trial	80,000 IU oral vitamin D_3_ either in the week following the suspicion or diagnosis of COVID‐19 or during the previous month	Control group	↓ Mortality, ↓ OSCI, ↑ Survival	Neutral
Zinc								
Yao et al. (2021)	USA	242 (138/104)	68	Quasi‐experimental trial	100 mg/d oral elemental zinc	Control group	↔ Mortality, ↔ Survival	Neutral
Derwand et al. (2020)	USA	518 (NR)	58	Quasi‐experimental trial	50 mg/d oral elemental zinc with 400 mg/d hydroxychloroquine and 500 mg/d azithromycin for 5 days	Control group	↓ Hospitalization, ↔ Mortality	Neutral
Carlucci et al. (2020)	USA	932 (584/348)	62	Quasi‐experimental trial	100 mg/d oral elemental zinc for 5 days	Control group	↔ Length of hospitalization, ↓ ICU admission, ↓ Ventilation requirement, ↔ Oxygen flow rate, ↔ FiO_2_, ↑ Discharged home	Neutral

*Note*: ↓, decrease; ↑, increase; ↔, no difference.

Abbreviations: BUN, blood urea nitrogen; Cr, creatinine; CRP, C‐reactive protein; d, day; FiO_2_, fraction of inspired oxygen; ICU, intensive care unit; IL‐6, interleukin‐6; IMVFD28, invasive mechanical ventilation‐free days in 28 days; IV, intravenous; MAP, mean arterial pressure; mo, month; NR, not reported; OSCI, ordinal scale for clinical improvement; PaO_2_, arterial oxygen partial pressure; PCT, procalcitonin; PT, prothrombin time; SOFA, sequential organ failure assessment; TBIL, total bilirubin; WBC, white blood cell count; wk, week.

^a^ Based on the Academy of Nutrition and Dietetics Quality Criteria Checklist (QCC) for Primary Research.

### 3.3 Vitamin A

For vitamin A, no observational and interventional studies had criteria for inclusion in this systematic review.

### 3.4 Vitamin C

For vitamin C, one observational (Arvinte et al., 2020) and two interventional (Hiedra et al., 2020; Zhang et al., 2021) studies were included in this systematic review. Interestingly, although no relationship was found between serum vitamin C levels and mortality in patients with COVID‐19 (Arvinte et al., 2020), intravenous supplementation with vitamin C significantly decreased mortality in severe cases (Zhang et al., 2021). In addition, vitamin C supplementation significantly reduced levels of inflammatory biomarkers such as interleukin‐6, ferritin, and D‐dimer in COVID‐19 patients (Hiedra et al., 2020; Zhang et al., 2021). Furthermore, supplementation with vitamin C caused a significant increase in Horowitz index, the ratio of arterial oxygen partial pressure to fractional inspired oxygen (Zhang et al., 2021). Nevertheless, vitamin C supplementation did not affect the length of hospitalization, disease complications, and some other clinical outcomes of subjects infected with SARS‐CoV‐2 (Hiedra et al., 2020; Zhang et al., 2021; Tables 1 and 2).

### 3.5 Vitamin D

For vitamin D, 20 observational (Abrishami et al., 2021; Bagheri et al., 2020; Baktash et al., 2021; Carpagnano et al., 2021; Daneshkhah et al., 2020; De Smet et al., 2021; Hars et al., 2020; Hernández et al., 2021; Karahan & Katkat, 2021; Laird et al., 2020; Luo et al., 2021; Maghbooli et al., 2020; Merzon et al., 2020; Padhi et al., 2020; Panagiotou et al., 2020; Pizzini et al., 2020; Radujkovic et al., 2020; Singh et al., 2020; Ye et al., 2021; Yılmaz & Şen, 2020) and four interventional (Annweiler, Corvaisier, et al., 2020; Annweiler, Hanotte, et al., 2020; Castillo et al., 2020; Rastogi et al., 2022) studies were included in this systematic review. Insufficient vitamin D levels were significantly related to the occurrence of COVID‐19 manifestations such as fever, hypoxia, lymphocytopenia, and unconsciousness (Maghbooli et al., 2020; Yılmaz & Şen, 2020). In addition, vitamin D status was significantly and inversely associated with lung involvement and ventilation requirement in COVID‐19 patients (Abrishami et al., 2021; Baktash et al., 2021). Moreover, although one study reported no relationship between dietary vitamin D intake and hospitalization (Bagheri et al., 2020), another study found a significant positive relationship between plasma vitamin D insufficiency and hospitalization (Merzon et al., 2020). Interestingly, a randomized controlled trial also showed that vitamin D supplementation significantly decreased intensive care unit (ICU) admission in individuals with COVID‐19 (Castillo et al., 2020). Furthermore, some observational studies detected a significant reverse relationship between vitamin D status and inflammatory biomarkers including C‐reactive protein (CRP), high‐sensitivity CRP, D‐dimer, serum ferritin, and neutrophil and lymphocyte counts (Daneshkhah et al., 2020; Hernández et al., 2021; Karahan & Katkat, 2021; Maghbooli et al., 2020; Pizzini et al., 2020); however, other observational studies did not (Hernández et al., 2020; Panagiotou et al., 2021; Pizzini et al., 2020). Likewise, the effect of oral vitamin D supplementation on inflammatory biomarkers was inconsistent in an interventional study of COVID‐19 patients (i.e., the positive effect on fibrinogen and neutral effect on others; Rastogi et al., 2022). Additionally, five studies found a significant protective role of vitamin D against the severe form of COVID‐19 (Bagheri et al., 2020; Luo et al., 2021; Maghbooli et al., 2020; Radujkovic et al., 2020; Ye et al., 2021), but one study did not (Hernández et al., 2021). Besides, most observational and interventional studies reported a beneficial role of vitamin D in favor of survival and against mortality in COVID‐19 patients (Abrishami et al., 2021; Annweiler, Hanotte, et al., 2020; Carpagnano et al., 2021; Daneshkhah et al., 2020; De Smet et al., 2021; Hars et al., 2020; Karahan & Katkat, 2021; Laird et al., 2020; Padhi et al., 2020; Radujkovic et al., 2020), whereas several studies did not find such a role (Annweiler, Hanotte, et al., 2020; Baktash et al., 2021; Hars et al., 2020; Panagiotou et al., 2020; Singh et al., 2020). Moreover, in two interventional studies with three treatment arms, supplementation with vitamin D significantly improved the Ordinal Scale for Clinical Improvement (OSCI) score of COVID‐19 patients in two treatment arms (Annweiler, Corvaisier, et al., 2020; Annweiler, Hanotte, et al., 2020) but not the third one (Annweiler, Hanotte, et al., 2020). The OSCI is a scale proposed by the WHO for assessment of COVID‐19 progression over time (World Health Organization, 2020). In spite of all the above, blood vitamin D status was not related to the length of hospitalization, chest radiological findings, and some other clinical outcomes of individuals infected with COVID‐19 (Baktash et al., 2020; Luo et al., 2021; Panagiotou et al., 2020; Tables 1 and 2).

### 3.6 Vitamin E

For vitamin E, no observational and interventional studies had criteria for inclusion in the present systematic review.

### 3.7 Selenium

For selenium, only two observational studies (Moghaddam et al., 2020; Jinsong Zhang et al., 2020) were included in the systematic review. In an ecological study, there was a significant positive relationship between mean levels of hair selenium and COVID‐19 cure rate, calculated as a percentage of the number of cured COVID‐19 patients over the number of confirmed cases (Jinsong Zhang et al., 2020). In a cross‐sectional research, serum levels of selenium, selenoprotein P, and glutathione peroxidase‐3 were negatively related to COVID‐19 mortality (Moghaddam et al., 2020; Table 1).

### 3.8 Zinc

For zinc, five observational (Anuk et al., 2021; Bagheri et al., 2020; Heller et al., 2020; Jothimani et al., 2020; Yasui et al., 2020) as well as three interventional (Carlucci et al., 2020; Derwand et al., 2020; Yao et al., 2021) studies were included in this systematic review. Two observational studies did not find any significant link between zinc status and COVID‐19 severity (Anuk et al., 2021; Bagheri et al., 2020), but one observational study reported a significant inverse relationship between serum levels of zinc and severity of COVID‐19 (Yasui et al., 2020). In addition, three studies did not detect a role for zinc in COVID‐19 survival and mortality (Derwand et al., 2020; Jothimani et al., 2020; Yao et al., 2021), but one study found a significant direct relationship between serum zinc levels and survival of subjects with COVID‐19 infection (Heller et al., 2020). Moreover, although zinc status was not significantly related to hospitalization and ICU admission (Bagheri et al., 2020; Jothimani et al., 2020), oral zinc sulfate supplementation significantly decreased hospitalization and ICU admission (Carlucci et al., 2020; Derwand et al., 2020). Zinc supplementation also caused a significant reduction in ventilation requirement and a significant increase in the number of COVID‐19 patients discharged from hospital to home (Carlucci et al., 2020). Furthermore, serum zinc levels were negatively associated with biomarkers of inflammation and bacterial infection including interleukin‐6, erythrocyte sedimentation rate, CRP, and procalcitonin (Anuk et al., 2021). Besides, there was a significant positive association between zinc deficiency and COVID‐19 complications (Jothimani et al., 2020). However, zinc did not change the length of hospitalization and some other clinical outcomes of people with COVID‐19 (Carlucci et al., 2020; Jothimani et al., 2020; Tables 1 and 2).

### 3.9 Α‐lipoic acid

For α‐lipoic acid, no observational and interventional studies had criteria for inclusion in this systematic review.

## 4 DISCUSSION

### 4.1 Summary of key findings

In this systematic review of primary human studies, we investigated the role of vitamins A, C, D, and E, selenium, zinc, and α‐lipoic acid in major clinical outcomes of people with COVID‐19. Among the aforementioned seven antioxidants, eligible studies were found only for vitamins C and D, selenium, and zinc. The findings suggest that vitamin C may cause beneficial effects on inflammation status, Horowitz index, and mortality rate of COVID‐19 patients. Moreover, vitamin D may have a positive role in the reduction of disease manifestations and severity, inflammatory biomarkers, lung involvement, ventilation requirement, hospitalization, ICU admission, and mortality in individuals with COVID‐19. Also, selenium may have the potential to increase and decrease the cure rate and mortality of COVID‐19 patients, respectively. Furthermore, zinc may be able to lower hospitalization, ventilation requirement, ICU admission, biomarkers of inflammation and bacterial infection, and disease complications in individuals infected with COVID‐19.

### 4.2 Mechanisms of actions

#### 4.2.1 Vitamin A

Although none of the included studies examined the role of vitamin A in subjects with COVID‐19, bioinformatics findings proposed that this antioxidant may be beneficial for individuals infected with SARS‐CoV‐2 (Li et al., 2020). Vitamin A has an important role in enhancing the body's immunity and regulating both cellular and humoral immune responses (Jayawardena et al., 2020). The production of antibodies, also known as immunoglobulins (Ig), is integral to the maintenance of humoral immune responses (Huang et al., 2018). An animal study showed that vitamin A can promote humoral immunity by increasing serum levels of IgG, IgM, and IgA (Ghodratizadeh et al., 2014). Vitamin A also plays a pivotal role in the development of epithelium, which is considered a frontline defense against pathogen invasion (McCullough et al., 1999). As vitamin A enhances mucin secretion in the respiratory tract and intestine, it is able to improve the antigen nonspecific immunity function of these tissues (Huang et al., 2018). Moreover, vitamin A may inhibit inflammatory processes induced by COVID‐19 through the regulation of multiple key genes including mitogen‐activated protein kinase 1 and 14, interleukin‐10, epidermal growth factor receptor, protein kinase C beta type, intercellular adhesion molecule 1, and catalase (Li et al., 2020).

#### 4.2.2 Vitamin C

The results of this systematic review indicated that vitamin C may exert favorable effects on clinical outcomes of COVID‐19 patients. Vitamin C acts as a powerful antioxidant, especially for epithelial cells of the lungs (Farjana et al., 2020). It appears to scavenge reactive oxygen species (ROS) and inhibit pathways involved in neutrophil extracellular trap formation and cytokine storms (Cerullo et al., 2020). Moreover, vitamin C can suppress lactate production. This can be of great importance because serum and tissue concentrations of lactate are elevated in critically ill patients with COVID‐19 (Earar et al., 2020). Lactate weakens the host immune system by decreasing the production of type I interferon and limiting viral clearance (Lottes et al., 2015; Zhang et al., 2019).

#### 4.2.3 Vitamin D

The findings of this systematic review showed that vitamin D may play a positive role in improvement of COVID‐19 clinical outcomes. It seems that antioxidative, antiinflammatory, and immunomodulatory properties of vitamin D can be involved in this regard (Hajhashemy et al., 2022; Musavi et al., 2020). Besides, some researchers discussed the key role of vitamin D in the RAS (Kumar et al., 2020; Malek Mahdavi, 2020; Musavi et al., 2020). As noted in the introduction, SARS‐CoV‐2 binds to ACE2, which is expressed on the surface of alveolar epithelial cells (Silvagno et al., 2020). Once the virus is attached, the activity of ACE2 is suppressed, which further enhances the activity of ACE1, that accordingly increases the formation of angiotensin II, leading to intensified pulmonary vasoconstriction and severe COVID‐19 reactions (Malek Mahdavi, 2020). In an animal study, the expression of ACE2 in the lungs was significantly elevated by calcitriol, the bioactive form of vitamin D (Xu et al., 2017). Therefore, as a result of vitamin D supplementation, ACE2 may be expressed more, which can decrease lung injury (Imai et al., 2005). Moreover, vitamin D may reduce the production of angiotensin II and result in less pulmonary vasoconstriction through suppressing renin activity (Kumar et al., 2020).

#### 4.2.4 Vitamin E

Although none of the included studies investigated the role of vitamin E in individuals with COVID‐19, bioinformatics findings suggested that this micronutrient may be beneficial for patients infected with SARS‐CoV‐2 (Kim et al., 2020). Vitamin E is a lipid‐soluble antioxidant with the ability to protect cells from damage caused by ROS, especially in respiratory infections (Lewis et al., 2019). Moreover, vitamin E is involved in various aspects of the immune response, including but not limited to the production of antibodies, phagocytosis, and T cell function (Akhtar et al., 2021). This vitamin modulates T cell function through affecting T cell membrane integrity, cell division, signal transduction, and several inflammatory mediators such as prostaglandin E_2_ and proinflammatory cytokines (Lewis et al., 2019). Furthermore, it seems that vitamin E can induce signals of gene expression that counteract signals associated with COVID‐19 (Kim et al., 2020).

#### 4.2.5 Selenium

The results of this systematic review revealed that selenium may have a promising role in amelioration of COVID‐19 clinical outcomes. As mentioned earlier, COVID‐19 increases the production of ROS in host cells, which can cause oxidative stress if not counteracted by the antioxidant defense system (Chernyak et al., 2020). Glutathione peroxidase‐1 (GPx1), a cytosolic selenoenzyme with antiviral properties, is considered as a crucial antioxidant defense against ROS (Sajjadi et al., 2022). This selenoprotein catalyzes the detoxification of hydrogen peroxide to water molecules and is particularly involved in protection against viral respiratory infections (Guillin et al., 2019). There is evidence of an interaction between GPx1 and the main protease of SARS‐CoV‐2, 3‐chymotrypsin‐like protease, which is essential for viral replication. This interaction depends on host selenium status to combat SARS‐CoV‐2 virulence (Seale et al., 2020). Accordingly, selenium may improve clinical outcomes of patients with COVID‐19.

#### 4.2.6 Zinc

The findings of this systematic review manifested that zinc may have desirable effects on clinical outcomes of COVID‐19 patients. Multiple protective mechanisms of zinc against COVID‐19 infection have been proposed in the literature. It seems that SARS‐CoV‐2 can weaken mucociliary clearance and expose the lungs to further viral and bacterial infections (Koparal et al., 2021). In turn, zinc may enhance mucociliary clearance by improving cilia morphology and increasing cilia beat frequency (Darma et al., 2020). This mineral can also improve the integrity and barrier function of the respiratory epithelium by increasing its antioxidant activity and upregulating its tight junction proteins such as claudin‐1 and zonula occludens‐1 (Skalny et al., 2020). In addition, zinc may exert antiviral effects through interference with viral replication cycles (Read et al., 2019). Moreover, zinc can be beneficial for bacterial coinfection in viral pneumonia, because it may inhibit the growth of *Streptococcus pneumoniae* by modulating bacterial manganese homeostasis (Eijkelkamp et al., 2019). Furthermore, zinc can downregulate the production of proinflammatory cytokines through the inhibition of IκB kinase activity and nuclear factor‐κB (NF‐κB) signaling (Skalny et al., 2020).

#### 4.2.7 Α‐lipoic acid

Although none of the included studies evaluated the role of α‐lipoic acid in patients with COVID‐19, some researchers hypothesized that this potent antioxidant may be advantageous for subjects infected with SARS‐CoV‐2 (Sayıner & Serakıncı, 2021). Α‐lipoic acid is able to reduce oxidative stress through the regeneration of other antioxidants and chelation of metal ions. In addition, this quasi‐vitamin can inhibit the activation of NF‐κB, an inflammatory transcription factor (Tibullo et al., 2017). Furthermore, α‐lipoic acid may decrease the activity of a disintegrin and metalloprotease 17 (ADAM17), also known as tumor necrosis factor‐α‐converting enzyme (Cure & Cure, 2020). The lower activity of ADAM17 can reduce the shedding of ACE2 and severity of COVID‐19 infection (Peron & Nakaya, 2020). Moreover, α‐lipoic acid may increase intracellular pH by activating Na^+^/K^+^‐ATPase (Cure & Cure, 2020). It seems that higher intracellular pH can inhibit SARS‐CoV‐2 cellular entry (Petersen et al., 2020). Also, α‐lipoic acid has a potential to activate pyruvate dehydrogenase and reduce serum lactate levels (Konrad et al., 1999).

### 4.3 Limitations

There are several limitations that need to be taken into consideration when interpreting the findings of this systematic review. First, no eligible studies were found for vitamins A and E as well as α‐lipoic acid. Second, a limited number of studies investigated the role of vitamin C, selenium, and zinc in clinical outcomes of COVID‐19 patients. In fact, most studies focused on the role of vitamin D. Third, most of the included records were observational studies with neutral quality. Fourth, the included studies were diverse in terms of study characteristics and methodology. Therefore, more high‐quality studies, especially randomized controlled trials, are required for future integration and consensus.

## 5 CONCLUSION

In conclusion, due to the important role of oxidative stress in the pathogenicity of SARS‐CoV‐2, antioxidants seem to be beneficial for patients with COVID‐19. Particularly, the findings obtained from this systematic review suggest that vitamins C and D, selenium, and zinc can improve some COVID‐19 clinical outcomes. Nevertheless, further well‐designed and well‐reported studies are needed to draw definite conclusions.

## CONFLICT OF INTEREST

None.

## ETHICAL STATEMENT

This study does not involve any human or animal testing.

## Supporting information


Table S1

## Data Availability

The data are available on request from the corresponding author.
