# Supplementary material for: Antioxidants and clinical outcomes of patients with coronavirus disease 2019: A systematic review of observational and interventional studies
Source: Food Sci Nutr. 2022 Sep 2;10(12):4112–25. doi: 10.1002/fsn3.3034 (PMC9538172; doi:10.1002/fsn3.3034)
Supplement: Supplementary file 1 — Table S1 [file FSN3-10-4112-s001.doc]

**Supplementary Table 1.** Detailed search strategy for each database or search engine.

| **Database or search engine** | **Search strategy** |
| --- | --- |
| Medline | ("vitamin A" [MeSH] OR "vitamin A" [TIAB] OR "retinoid" [MeSH] OR "retinoid" [TIAB] OR "retinol" [MeSH] OR "retinol" [TIAB] OR "retinal" [MeSH] OR "retinal" [TIAB] OR "retinoic acid" [MeSH] OR "retinoic acid" [TIAB] OR "retinyl ester" [MeSH] OR "retinyl ester" [TIAB] OR "aquasol A" [MeSH] OR "aquasolA" [TIAB] OR "vitamin C" [MeSH]OR "vitamin C" [TIAB] OR "ascorbic acid" [MeSH] OR "ascorbic acid" [TIAB] OR "ascorbate" [MeSH] OR "ascorbate" [TIAB] OR "vitamin D" [MeSH] OR "vitamin D" [TIAB] OR "cholecalciferol" [MeSH] OR "cholecalciferol" [TIAB] OR "colecalciferol" [MeSH] OR "colecalciferol" [TIAB] OR "ergocalciferol" [MeSH] OR "ergocalciferol" [TIAB] OR "calciferol" [MeSH] OR "calciferol" [TIAB] OR "hydroxycholecalciferol" [MeSH] OR "hydroxycholecalciferol" [TIAB] OR "calcifediol" [MeSH] OR "calcifediol" [TIAB] OR "calcidiol" [MeSH] OR "calcidiol" [TIAB] OR "25-hydroxycholecalciferol" [MeSH] OR "25-hydroxycholecalciferol" [TIAB] OR "25-hydroxyvitamin D" [MeSH] OR "25-hydroxyvitamin D" [TIAB] OR "calcitriol" [MeSH] OR "calcitriol" [TIAB] OR "1,25-dihydroxycholecalciferol" [MeSH] OR "1,25-dihydroxycholecalciferol" [TIAB] OR "1,25-dihydroxyvitamin D" [MeSH] OR "1,25-dihydroxyvitamin D" [TIAB] OR "alfacalcidol" [MeSH] OR "alfacalcidol" [TIAB] OR "1-alpha-hydroxyvitamin D" [MeSH] OR "1-alpha-hydroxyvitamin D" [TIAB] OR "1-α-hydroxyvitamin D" [MeSH] OR "1-α-hydroxyvitamin D" [TIAB] OR "paricalcitol" [MeSH] OR "paricalcitol" [TIAB] OR "vitamin E" [MeSH] OR "vitamin E" [TIAB] OR "tocopherol" [MeSH] OR "tocopherol" [TIAB] OR "tocotrienol" [MeSH] OR "tocotrienol" [TIAB] OR "selenium" [MeSH] OR "selenium" [TIAB] OR "selenite" [MeSH] OR "selenite" [TIAB] OR "selenate" [MeSH] OR "selenate" [TIAB] OR "Se" [MeSH] OR "Se" [TIAB] OR "zinc" [MeSH] OR "zinc" [TIAB] OR "Zn"[MeSH] OR"Zn" [TIAB] OR "lipoic acid" [MeSH] OR "lipoic acid" [TIAB] OR"α-lipoic acid" [MeSH] OR "α-lipoic acid" [TIAB] OR "alpha-lipoic acid" [MeSH] OR "alpha-lipoic acid" [TIAB] OR "thioctic acid" [MeSH] OR "thioctic acid" [TIAB]) AND ("coronavirus disease 2019"[MeSH]OR "coronavirus disease 2019" [TIAB] OR "coronavirus disease-19" [MeSH] OR "coronavirus disease-19" [TIAB] OR"COVID-19" [MeSH] OR "COVID-19" [TIAB] OR "2019 novel coronavirus" [MeSH] OR "2019 novel coronavirus" [TIAB] "2019-nCoV" [MeSH] OR "2019-nCoV" [TIAB] OR "severe acute respiratory syndrome coronavirus 2" [MeSH] OR "severe acute respiratory syndrome coronavirus 2" [TIAB] OR "SARS-CoV-2" [MeSH] OR "SARS-CoV-2" [TIAB] OR "Wuhan coronavirus" [MeSH] OR "Wuhan coronavirus" [TIAB]) |
| Scopus | (TITLE-ABS-KEY("vitamin A") OR TITLE-ABS-KEY ("retinoid") OR TITLE-ABS-KEY ("retinol") OR TITLE-ABS-KEY ("retinal") OR TITLE-ABS-KEY ("retinoic acid") OR TITLE-ABS-KEY("retinyl ester") OR TITLE-ABS-KEY("aquasol A") OR TITLE-ABS-KEY("vitamin C") OR TITLE-ABS-KEY("ascorbic acid") OR TITLE-ABS-KEY("ascorbate") OR TITLE-ABS-KEY("vitamin D") OR TITLE-ABS-KEY("cholecalciferol") OR TITLE-ABS-KEY("colecalciferol") OR TITLE-ABS-KEY("ergocalciferol") OR TITLE-ABS-KEY("calciferol") OR TITLE-ABS-KEY("hydroxycholecalciferol") OR TITLE-ABS-KEY("calcifediol") OR TITLE-ABS-KEY("calcidiol") OR TITLE-ABS-KEY("25-hydroxycholecalciferol") OR TITLE-ABS-KEY("25-hydroxyvitamin D") OR TITLE-ABS-KEY("calcitriol") OR TITLE-ABS-KEY("1,25-dihydroxycholecalciferol") OR TITLE-ABS-KEY("1*,*25*-*dihydroxyvitamin D") OR TITLE-ABS-KEY("alfacalcidol") ORTITLE-ABS-KEY("1-alpha-hydroxyvitamin D") OR TITLE-ABS-KEY("1-α-hydroxyvitamin D") OR TITLE-ABS-KEY("paricalcitol") OR TITLE-ABS-KEY("vitamin E") OR TITLE-ABS-KEY("tocopherol") OR TITLE-ABS-KEY("tocotrienol") OR TITLE-ABS-KEY("selenium") OR TITLE-ABS-KEY("selenite") OR TITLE-ABS-KEY("selenate") OR TITLE-ABS-KEY("Se") OR TITLE-ABS-KEY("zinc") OR TITLE-ABS-KEY("Zn") OR TITLE-ABS-KEY("lipoic acid") OR TITLE-ABS-KEY("α-lipoic acid") OR TITLE-ABS-KEY("alpha-lipoic acid") OR TITLE-ABS-KEY("thioctic acid")) AND (TITLE-ABS-KEY("coronavirus disease 2019") OR TITLE-ABS-KEY("coronavirus disease-19") OR TITLE-ABS-KEY("COVID-19") OR TITLE-ABS-KEY("2019 novel coronavirus") OR TITLE-ABS-KEY("2019-nCoV") OR TITLE-ABS-KEY ("severe acute respiratory syndrome coronavirus 2") OR TITLE-ABS-KEY("SARS-CoV-2") OR TITLE-ABS-KEY ("Wuhan coronavirus")) |
| Web of Science | (TS=("vitamin A") OR TS=("retinoid") OR TS=("retinol") OR TS=("retinal") OR TS=("retinoic acid") OR TS=("retinyl ester") OR TS=("aquasol A") OR TS=("vitamin C") OR TS=("ascorbic acid") OR TS=("ascorbate") OR TS=("vitamin D") OR TS=("cholecalciferol") OR TS=("colecalciferol") OR TS=("ergocalciferol") OR TS=("calciferol") OR TS=("hydroxycholecalciferol") OR TS=("calcifediol") OR TS=("calcidiol") OR TS=("25-hydroxycholecalciferol") OR TS=("25-hydroxyvitamin D") OR TS=("calcitriol") OR TS=("1,25-dihydroxycholecalciferol") OR TS=("1,25-dihydroxyvitamin D") OR TS=("alfacalcidol") OR TS=("1-alpha-hydroxyvitamin D") OR TS=("1-α-hydroxyvitamin D") OR TS=("paricalcitol") OR TS=("vitamin E") OR TS=("tocopherol") OR TS=("tocotrienol") OR TS=("selenium") OR TS=("selenite") OR TS=("selenate") OR TS=("Se") OR TS=("zinc") OR TS=("Zn") OR TS=("lipoic acid") OR TS=("α-lipoic acid") OR TS=("alpha-lipoic acid") OR TS=("thioctic acid")) AND (TS=("coronavirus disease 2019") OR TS=("coronavirus disease-19") OR TS=("COVID-19") OR TS=("2019 novel coronavirus") OR TS=("2019-nCoV") OR TS=("severe acute respiratory syndrome coronavirus 2") OR TS=("SARS-CoV-2") OR TS=("Wuhan coronavirus")) |
| Cochrane Library | ("vitamin A" OR "retinoid" OR "retinol" OR "retinal" OR "retinoic acid" OR "retinyl ester" OR "aquasol A" OR "vitamin C" OR "ascorbic acid" OR "ascorbate" OR "vitamin D" OR "cholecalciferol" OR "colecalciferol" OR "ergocalciferol" OR "calciferol" OR "hydroxycholecalciferol" OR "calcifediol" OR "calcidiol" OR "25-hydroxycholecalciferol" OR "25-hydroxyvitamin D" OR "calcitriol" OR "1,25-dihydroxycholecalciferol" OR "1,25-dihydroxyvitamin D" OR "alfacalcidol" OR "1-alpha-hydroxyvitamin D" OR "1-α-hydroxyvitamin D" OR "paricalcitol" OR "vitamin E" OR "tocopherol" OR "tocotrienol" OR "selenium" OR "selenite" OR "selenate" OR "Se" OR "zinc" OR "Zn" OR "lipoic acid" OR "α-lipoic acid" OR "alpha-lipoic acid" OR "thioctic acid") AND ("coronavirus disease 2019" OR "coronavirus disease-19" OR "COVID-19" OR "2019 novel coronavirus" OR "2019-nCoV" OR "severe acute respiratory syndrome coronavirus 2" OR "SARS-CoV-2" OR "Wuhan coronavirus") |
| Google Scholar | ("vitamin A" OR "retinoid" OR "retinol" OR "retinal" OR "retinoic acid" OR "retinyl ester" OR "aquasol A" OR "vitamin C" OR "ascorbic acid" OR "ascorbate" OR "vitamin D" OR "cholecalciferol" OR "colecalciferol" OR "ergocalciferol" OR "calciferol" OR "hydroxycholecalciferol" OR "calcifediol" OR "calcidiol" OR "25-hydroxycholecalciferol" OR "25-hydroxyvitamin D" OR "calcitriol" OR "1,25-dihydroxycholecalciferol" OR "1,25-dihydroxyvitamin D" OR "alfacalcidol" OR "1-alpha-hydroxyvitamin D" OR "1-α-hydroxyvitamin D" OR "paricalcitol" OR "vitamin E" OR "tocopherol" OR "tocotrienol" OR "selenium" OR "selenite" OR "selenate" OR "Se" OR "zinc" OR "Zn" OR "lipoic acid" OR "α-lipoic acid" OR "alpha-lipoic acid" OR "thioctic acid") AND ("coronavirus disease 2019" OR "coronavirus disease-19" OR "COVID-19" OR "2019 novel coronavirus" OR "2019-nCoV" OR "severe acute respiratory syndrome coronavirus 2" OR "SARS-CoV-2" OR "Wuhan coronavirus") |
